# Imaging Modality Selection in Cardiac Ablation

**DOI:** 10.19102/icrm.2022.130402

**Published:** 2022-04-15

**Authors:** Christian Ngo, Nazem Akoum

**Affiliations:** ^1^Department of Cardiology, University of Washington School of Medicine, Seattle, WA, USA

**Keywords:** Cardiac CT, cardiac MRI, intracardiac echocardiography, nuclear imaging

## Abstract

Catheter ablation as a treatment method for both ventricular and atrial arrhythmias has evolved significantly over the past 40 years since it was first performed in humans. This evolution has been paralleled by a similar expansion in both invasive and non-invasive imaging modalities directed at further elucidating cardiac morphology and arrhythmia substrate pathophysiology. Access to multimodality imaging options is a significant piece of the armamentarium available to interventional electrophysiologists who are tackling increasingly complex rhythm problems with catheter ablation. This presents a unique problem to the practicing electrophysiologist in selecting the most pertinent imaging modalities that will improve the safety and efficacy of a procedure and winnowing out potential imaging studies that offer minimal or marginal benefit. In this review, we evaluate the various modalities that are useful in planning and executing successful ablation and weigh the evidence for benefit.

## Introduction

Catheter ablation as a treatment method for both ventricular and atrial arrhythmias has evolved significantly over the past 40 years since it was first performed in humans.^1,2^ This evolution has been paralleled by a similar expansion in both invasive and non-invasive imaging modalities directed at further elucidating cardiac morphology and arrhythmia substrate pathophysiology.^3^ Access to multimodality imaging options is a significant piece of the armamentarium available to interventional electrophysiologists who are tackling increasingly complex rhythm problems with catheter ablation. This presents a unique problem to the practicing electrophysiologist in selecting the most pertinent imaging modalities that will improve the safety and efficacy of a procedure and winnowing out potential imaging studies that offer minimal or marginal benefit. In this review, we evaluate the various modalities that are useful in planning and executing successful ablation and weigh the evidence for benefit.

## Catheter ablation for atrial fibrillation

### Preprocedural imaging

Multimodality imaging has emerged as a useful tool in the preprocedural assessment for atrial fibrillation (AF) ablation. Cardiac magnetic resonance imaging (MRI), computed tomography (CT), and echocardiography can be used to help anticipate and plan for technical difficulties due to left atrial and pulmonary vein structural variation as well as aid in the prognostication of long-term success in maintaining sinus rhythm after ablation.

Left atrial appendage thrombus is a contraindication to the AF ablation procedure. Traditionally, evaluation for left atrial appendage thrombus has been done with transesophageal echocardiography. This is widely accepted as the gold-standard test and the modality recommended for left atrial thrombus evaluation in both the European Society of Cardiology and American College of Cardiology AF guidelines with a sensitivity of 93%–100% and a specificity of 99%–100%.^4–7^ However, cardiac CT has emerged as a highly sensitive and specific test for the exclusion of left atrial thrombus, with a meta-analysis showing a sensitivity of 96% and a negative predictive value of 99% tempered by a low overall positive predictive value of 41%.^8^ This can be improved by the performance of delayed-phase CT, which improves the sensitivity to 100% and specificity to 99%, with the positive predictive value increasing to 92% and the negative predictive value increasing to 100%.^8^ With this level of diagnostic accuracy, cardiac CT could be considered in lieu of transesophageal echocardiogram to rule out left atrial appendage thrombus. However, limitations of cardiac CT include the requirement for iodinated contrast and ionizing radiation, and delayed-phase CT increases the radiation exposure associated with this approach. Cardiac MRI can also be used to evaluate for left atrial appendage thrombus with high accuracy, though this is impacted by the type of sequences performed. Studies looking at this question in cardiac MRI have been limited, and a meta-analysis found that cardiac MRI in general had an 80% sensitivity and a 99% specificity for left atrial thrombus.^9^ Contrast-enhanced and late gadolinium enhancement (LGE) sequences can improve the specificity of this imaging modality.^10^

**Figure 1:**
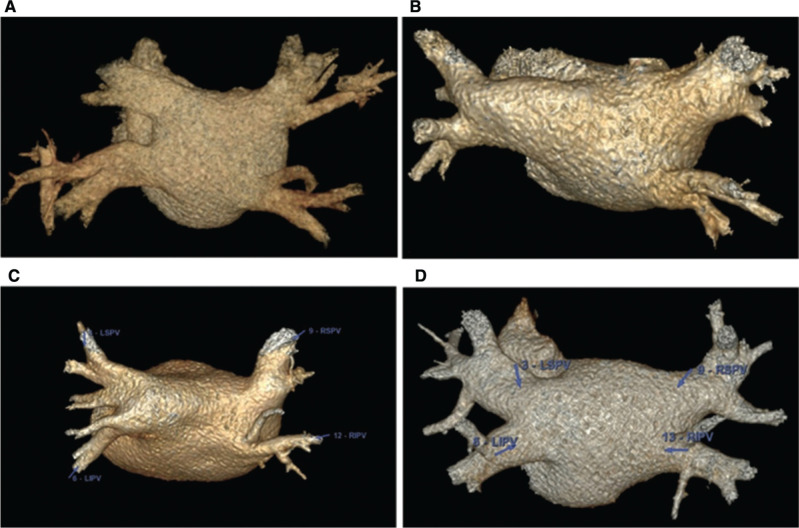
Cardiac computed tomography demonstrating variations in pulmonary venous anatomy. **A**: Two left pulmonary veins and 2 right pulmonary veins. **B:** One common left pulmonary vein and 2 right pulmonary veins. **C:** Two left pulmonary veins with early branching and 2 right pulmonary veins. **D:** Two left pulmonary veins and 3 right pulmonary veins.

Completing a structural assessment of the pulmonary veins, including pulmonary vein ostial size and variant pulmonary venous return and branching, prior to ablation is extremely important, in particular when planning for balloon-based procedures like cryoablation where the pulmonary venous anatomy may have a significant impact on durable procedural success. A significant amount of variation exists in the pulmonary venous anatomy **(Figure 1)**. While there is significant variation in the literature available, some studies have shown that patients with variant pulmonary venous anatomy may have different procedural efficacies compared to those with typical anatomy. One study reported a decrease in the success of cryoablation in patients with a left common pulmonary vein, while other studies have found that patients with normal right-sided pulmonary vein anatomy had a high risk of recurrence of AF after radiofrequency (RF) ablation.^11,12^ Still, others have found no difference in procedural success outcomes between cryoballoon ablation and RF ablation in patients with variant pulmonary venous anatomy.^13^

Left atrial size as assessed by echocardiography has been associated with the recurrence of AF after catheter ablation.^12,14^ Normal values of left atrial volume have been established in cardiac MRI^15^ as well as in cardiac CT^16^ studies. Increased left atrial volume on both cardiac CT and cardiac MRI scans has been associated with an increased risk of recurrence after AF ablation, though clear threshold values of the left atrial size that indicate that a very high risk of recurrence has not been established.^17,18^ While all 3 modalities can measure left atrial dimensions, it is important to note that there is significant variation between modality measurements in the same population, with transthoracic echocardiography tending to underestimate the left atrial volume more than cardiac MRI and cardiac CT.^19^ In addition to its impact on potential procedural success, left atrial size may also have an impact on the ablation modality that one chooses. While cryoballoon ablation has been found to be effective with shorter procedural times than RF ablation, patients with significantly dilated atria may have an increased risk of AF recurrence after cryoballoon ablation as opposed to RF ablation.^20^

**Figure 2:**
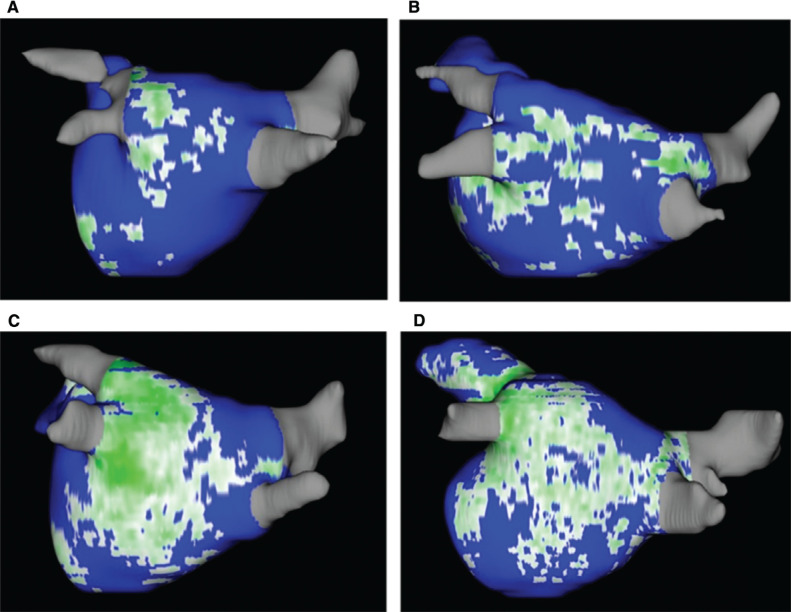
Cardiac magnetic resonance imaging of the left atrium with postprocessing of late gadolinium enhancement sequences depicting a left atrial scar. The scar is quantified and patients can be categorized by their degree of fibrosis. **A:** Utah class I—5.1% fibrosis. **B:** Utah class II—18.2% fibrosis. **C:** Utah class III—24.5% fibrosis. **D:** Utah class III—35.4% fibrosis.

Cardiac MRI as a preprocedural imaging modality has an advantage over cardiac CT and transesophageal echocardiography in that it provides not only non-invasive and radiation-free anatomic characterization but also tissue characterization of the left atrium through the evaluation of fibrosis. This functional characterization has prognostic implications when counseling patients regarding the likelihood of durable success. Increased LGE uptake in the left atrium has been associated with a greater likelihood of recurrent atrial arrhythmia after AF ablation in both long-term and short-term follow-up studies.^21–23^ Evaluation of AF with LGE-MRI is increasing but remains limited to specialized centers with expertise in image acquisition of the thin-walled left atrium and image processing to quantify fibrosis.^24^ Examples of degrees of atrial fibrosis can be seen in **Figure 2**. Integration of machine-learning algorithms with LGE-MRI data has been used to predict recurrence after AF ablation and, while these methodologies are still in their infancy, they appear to have potential.^25^ LGE-MRI prior to catheter ablation has also been used to generate computational simulations to identify AF reentrant drivers. Targeting these driver areas has been demonstrated to improve catheter ablation outcomes.^26^

### Intraprocedural imaging

Preprocedural cross-sectional imaging with cardiac MRI and cardiac CT as well as intraprocedural intracardiac echocardiography (ICE) can now be widely integrated into electroanatomic mapping (EAM) systems during AF ablation. Image integration has been shown to improve procedural outcomes in AF ablation.^27^ In addition, utilization of image integration has been shown to decrease both overall procedural times as well as fluoroscopy utilization.^28^

ICE has become an integral part of AF ablation procedures in the United States. It is useful not only in the performance of the transseptal puncture but also in the assessment of the anatomy of the pulmonary veins and for the evaluation of baseline and procedure-associated pericardial effusion. Real-time three-dimensional (3D) ICE has also become more widely available and allows for better visualization of cardiac structures from angles that may not be available with traditional ICE manipulation.^29^

Zero-fluoroscopy or minimal-fluoroscopy AF ablation has also recently been highlighted as a means of decreasing radiation exposure to both the patient and the operator and staff during the procedure. These align with the goal to achieve radiation doses “as low as reasonably achievable.” Several studies have been published reviewing the technique for achieving zero-fluoroscopy in all aspects of AF ablation, including coronary sinus catheter cannulation, transseptal puncture, EAM, and ablation.^30–33^ A complete primer on these techniques goes beyond the scope of this review, but they rely heavily on ICE, integration with preprocedural CT or MRI imaging, and EAM. In the comparisons of zero-fluoroscopy or minimal-fluoroscopy techniques with standard fluoroscopically guided AF ablation, procedural times appear to be similar, and zero-fluoroscopy ablation is feasible in most patients.^32,34^ Importantly, among experienced operators, overall efficacy and procedural complications appear to be similar to those reported for fluoroscopically guided AF ablation.^35,36^ Zero-fluoroscopy techniques have also been shown to be feasible in patients undergoing cryoablation for AF, though more extensive studies have not yet been conducted.^37^

### Postprocedural imaging

**Figure 3:**
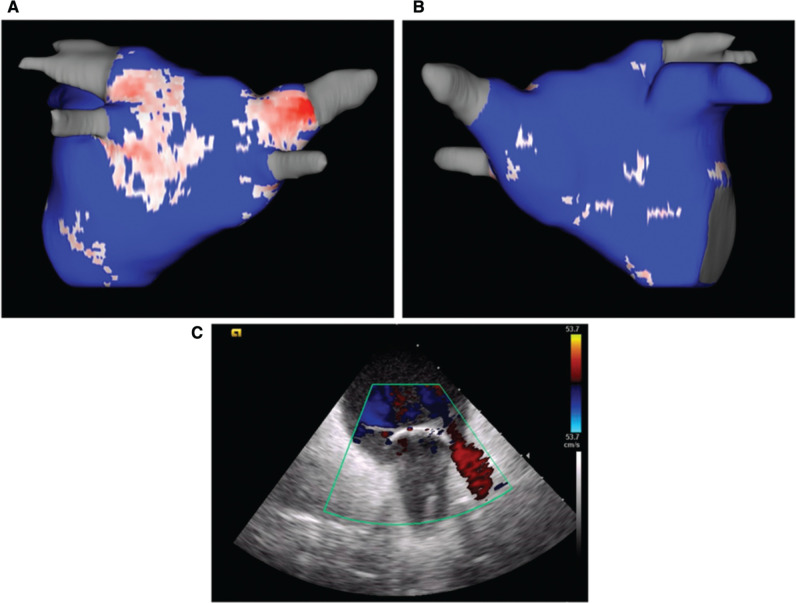
Postablation left atrial fibrosis map showing a postablative scar in red on the posterior (**A**) and anterior (**B**) left atrial walls with gaps on the anterior wall corresponding to an area of incomplete seal of the cryoablation balloon seen on intracardiac echocardiography images (**C**). Subsequent repeat ablation showed leak anteriorly with successful ablation in this area for pulmonary vein reisolation.

LGE-MRI provides a unique look into the pathophysiological underpinnings of AF and atrial tachycardia and its recurrence. For patients with recurrence of AF, a postablation LGE scar can be indicative of areas most likely to have had inadequate ablation and can direct repeat catheter ablation to areas of reconnection, as seen in **Figure 3**. In addition, catheter ablation, with the goal of homogenizing an existing atrial scar, can be a useful strategy in preventing arrhythmia recurrence.^38^ For patients with macro-reentrant tachycardia, while EAM-guided ablation is the conventional strategy used, LGE-MRI-guided ablation targeting areas of non-transmural scarring and channels or gaps may yield better long-term results.^38^

In addition, the existence of any postablative pulmonary vein stenosis is an important factor to consider. The incidence of pulmonary vein stenosis after AF ablation is highly variable depending upon the era studied, but more recent estimates suggest that the incidence of pulmonary vein stenosis is in the 3%–8% range.^39^ Having baseline pulmonary vein dimensions is critical to potentially avoiding further ostial ablation lesions in affected veins.

## Supraventricular tachycardia and typical atrial flutter ablation

While multimodality preprocedural imaging is not standard for all patients undergoing supraventricular tachycardia or typical atrial flutter ablation, it can be extremely useful in special patient populations. In patients with structural heart disease, preprocedural imaging with echocardiography is an important factor in counseling patients about the risks of procedural sedation and ablation as well as understanding the potential need for deflectable sheaths or larger-curve catheters in patients with significantly enlarged atria. In patients with congenital heart disease, cardiac MRI and cardiac CT are useful to delineate the congenital lesion and surgical corrections as well as anomalies of the vasculature that may be relevant to ablation.

**Figure 4:**
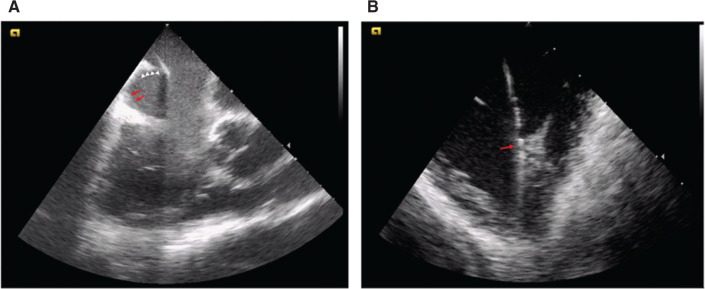
Intracardiac ultrasound can be an indispensable tool in navigating complex atrial and ventricular anatomy. **A:** Prominent Eustachian ridge (white arrowheads) impeding catheter access to the cavotricuspid isthmus and requiring a “candy cane” approach with the ablation catheter (red arrow). **B:** Patient with symptomatic premature ventricular complexes (PVCs) that were only terminated by ablation at the bifurcation of the right ventricular papillary muscle (red arrow) seen with intracardiac echocardiography. Ablation lesions delivered on either site of the structure led to partial suppression but not complete elimination of the PVC.

Intraprocedural imaging with ICE can be particularly useful in patients undergoing typical atrial flutter ablation. The cavotricuspid isthmus is a complex structure with significant variation in anatomy, including the Eustachian ridge size and the presence of tissue pouches.^40^ In cases of previously failed typical flutter ablation, ICE imaging has proven useful in identifying either a large pouch or a prominent Eustachian ridge as the anatomical barrier, and this knowledge has contributed to successful ablation^41^ **(Figure 4A)**. In a small randomized study, use of ICE in atrial flutter ablation was associated with decreased fluoroscopy time and improved procedural success but not with reductions in ablation time or procedural duration.^42^ In addition, in patients with congenital heart disease, tricuspid valve disease, or valve replacement, ICE images are useful in guiding baffle punctures, identifying potential anatomic barriers to successful ablation as well as guiding safe and effective trajectories for catheter motion. As with AF ablation, zero-fluoroscopy techniques have been shown to be safe and effective in supraventricular tachycardia and atrial flutter ablation. These techniques rely heavily on the integration of ICE with the EAM system to guide ablation, as described earlier.

## Ventricular arrhythmia ablation

### Preprocedural imaging

Preprocedural imaging in patients referred for ventricular tachycardia (VT) or premature ventricular complex (PVC) ablation can be critical in determining the success of an ablation-based strategy and guiding the operator toward areas of interest for ablation. Contrast-enhanced cardiac MRI has emerged as the dominant imaging modality in this patient population primarily for its ability to delineate areas of myocardial scar and the border zone. However, postprocessing advancement in delayed-enhancement cardiac CT has been shown to be potentially useful in this area as well. In addition, cardiac positron emission tomography is useful in the evaluation of potential inflammatory cardiomyopathies as medical therapy approaches may be more prudent in the active inflammatory phase.^43^

Cardiac MRI has a strong diagnostic role in the evaluation of patients with ventricular arrhythmias. In up to 67% of survivors of sudden cardiac arrest with an inconclusive diagnosis, cardiac MRI revealed evidence of structural abnormalities and, in 38% of patients, led to a new or alternate diagnosis compared to a routine workup.^44^ In patients with idiopathic PVCs or VT, cardiac MRI can also visualize areas of scarring during an otherwise normal structural evaluation on cardiac echocardiographs. One study evaluating patients with idiopathic left ventricular (LV) outflow tract PVCs compared to non-LVOT PVCs reported a higher prevalence of basal septal LGE.^45^ In addition, those with septal LGE were more likely to experience VT recurrence with a similar morphology after successful ablation compared to those who did not have LGE.^45^

Cardiac MRI can help guide EAM and delineate areas of potential substrate for ablation targeting.^46^ Preprocedural cardiac MRI has been associated with improved VT ablation success as well as long-term outcomes.^47^ A recent meta-analysis of preprocedural image–guided VT ablation compared to non–image-guided ablation showed no significant difference in the procedural time but documented a statistically significant improvement in VT-free survival as well as overall survival.^48^ Preprocedural cardiac MRI can be a useful prognosticator for patients undergoing VT ablation. In a study of ischemic VT patients, a larger scar as measured by LGE-MRI was associated with clinical recurrence after catheter ablation.^49^ This correlated well with EAM-defined scar areas and had a higher predictive accuracy compared to scar extent by EAM. In idiopathic dilated cardiomyopathy, 2 distinct groupings of scar have been seen on LGE that correlate with scar on EAM, including predominantly anteroseptal scar and predominantly inferolateral scar.^50^ Patients with anteroseptal scarring are at higher risk of recurrence of VT in the long term and are more likely to require repeat ablation.^51^ In addition, a non-ischemic anteroseptal scar often extends to involve the LV endocardium and epicardium around the aortic valve plane and, given the proximity of the scar to the native conduction system, there is elevated concern of the need for permanent pacing after ablation.^52^

Preprocedural MRI can also be utilized to guide ablation and focus EAM in areas of the heart where critical isthmus sites for VT are most likely to be found. While surface electrocardiography (ECG) criteria have traditionally been used to guide mapping and the site of origin, there are many disadvantages to surface ECG for localization, in particular in patients with a large or heterogeneous myocardial scar. One critical question where surface ECG patterns are often utilized to predict the outcome is the likelihood of a critical epicardial component. LGE-MRI can be used as an adjunctive measure in determining the predominant scar pattern and the likelihood of successful ablation targets being in the epicardium versus the endocardium. The existence of an epicardial scar and, in particular, a predominance of epicardial scarring over mid-myocardial or endocardial scars, has been shown to predict the need for epicardial ablation.^53^ This has been corroborated by Njeim et al. in patients who are undergoing redo VT ablation, with those demonstrating an epicardial scar on LGE-MRI being more likely to have epicardial substrate and requiring epicardial ablation.^54^ This is extremely important to know up-front for procedural planning, as this significantly impacts the workflow of the case and could require referral to a different facility with expertise in epicardial access and ablation.

**Figure 5:**
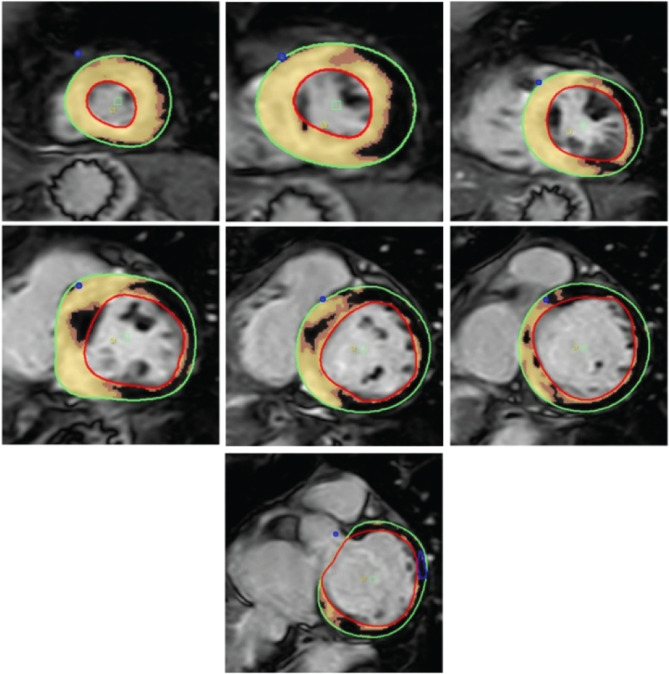
Cardiac magnetic resonance imaging of a patient with a history of hypertrophic cardiomyopathy and VT demonstrating the complexity of the myocardial substrate and the potential need for epicardial ablation. A dense scar with high signal intensity is delineated in yellow, and the scar border zone is delineated in orange.

Beyond the prediction of the need for epicardial ablation, the ablation target site can also be further elucidated with preprocedural imaging. In non-ischemic patients, Kuo et al. found that the critical site for septal VT occurred in areas of high signal intensity on LGE-MRI^55^ **(Figure 5)**. These were more likely to be close to the aortic valve plane in patients with idiopathic dilated cardiomyopathy. Anteroseptal scar extent on LGE imaging has also been shown to have a potential prognostic role, with patients showing full-length septal LGE having a significantly higher recurrence rate of VT than those with only partial-length septal LGE.^56^ Delayed-enhancement border zone areas have good correlation with conduction channels visualized through EAM,^57^ and these border zone areas can identify the critical isthmus for VT circuits in up to 74% of cases.^58^ In both ischemic and non-ischemic cardiomyopathy, MRI-derived core-border zone transition and >75% transmural scars contained a majority of critical isthmus sites for VT in a prior study.^59^

For patients with myocarditis undergoing VT ablation due to medication-refractory VT, active myocarditis using the MRI Lake Louise Criteria or by endomyocardial biopsy has been associated with longer-term VT recurrence.^60^ It is suggested that these patients may not benefit from VT ablation and should be managed medically if possible until they enter the postmyocarditis phase.

**Figure 6:**
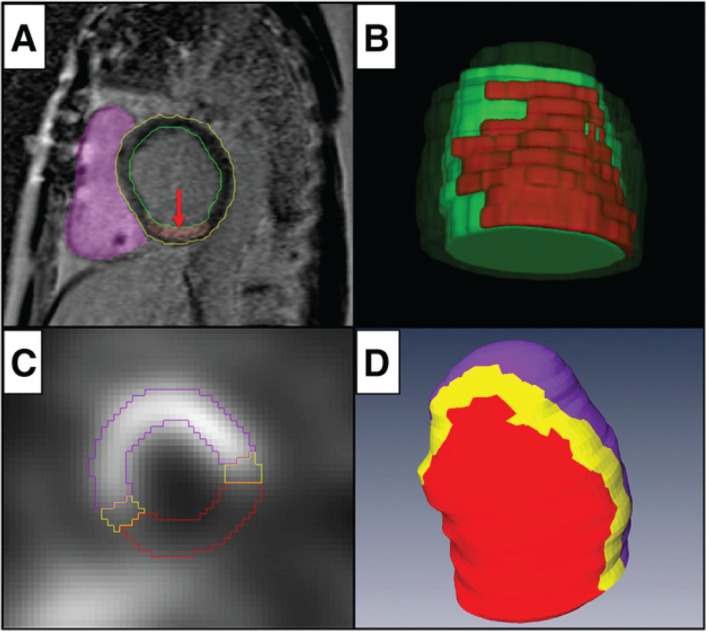
Late-gadolinium enhancement (LGE) cardiac magnetic resonance (CMR) and ^123^I-metaiodobenzylguanidine (MIBG) single-photon emission computerized tomography (SPECT) reconstruction. **A:** Epicardial (yellow line) and endocardial (green line) borders of a left ventricle outlined by manual segmentation of short-axis LGE CMR slices. Scar reconstruction was based on voxel intensity (red arrow). **B:** Three-dimensional reconstruction of magnetic resonance imaging (MRI)-based myocardial scar (red) embedded in myocardial reconstruction (green). **C:** ^123^I-MIBG SPECT short-axis slice demonstrating a lack of uptake in the inferolateral wall, consistent with abnormal innervation. **D:** Three-dimensional reconstruction of ^123^I-MIBG SPECT innervation map with inferior view demonstrating normally innervated myocardium (purple), abnormally innervated myocardium (red; 50% uptake), and transition zone myocardium (yellow). Reprinted with permission from Imanli H, Ume KL, Jeudy J, et al. Ventricular tachycardia (VT) substrate characteristics: insights from multimodality structural and functional imaging of the VT substrate using cardiac MRI scar, ^123^I-metaiodobenzylguanidine SPECT innervation, and bipolar voltage. *J Nucl Med*. 2019;60(1):79–85.

^123^I-metaiodobenzylguanidine (MIBG) single-photon emission computerized tomography (SPECT) has been used as an adjunct to EAM and cardiac MRI to help delineate scarring. ^123^I-MIBG SPECT is used to image areas of sympathetic innervation to the heart. This adds an additional layer of functional assessment of the myocardium above the structural assessment provided by cardiac MRI seen in **Figure 6**. In one study, a VT channel and exit sites were found to be localized to areas with both cardiac MRI LGE and ^123^I-MIBG SPECT abnormal innervation.^61^ Preprocedural cardiac MRI and cardiac CT have also been used to guide transarterial coronary ethanol ablation in a small case series.^62^

Recent studies have also demonstrated the potential of imaging-based computational simulations to guide catheter ablation by identifying potential targets. These methods, as described by Trayanova et al., use finite element models derived from LGE-MRI sequences to simulate pacing maneuver–induced arrhythmogenicity in the model ventricle.^63^ These methods have been utilized to accurately predict ablation targets found during EAM in 9 of 11 patients with primarily ischemic VT in the study by Ashikaga et al.^64^

### Intraprocedural imaging

Intraprocedural imaging for VT ablation primarily involves 2 major aspects: image integration into the EAM system and real-time imaging during ablation. Integration of contrast-enhanced cardiac MRI into the EAM has been shown to increase the likelihood of both non-inducibility with substrate ablation and freedom from VT recurrence in non-randomized studies. Andreu et al. described an imaging-guided ablation strategy in which the MRI is processed into 5 concentric layers from the endocardium to the epicardium in a semiautomatic fashion.^65^ The cardiovascular magnetic resonance (CMR) information was then projected onto each surface. Regions of scar core, border zone, and healthy tissue were then assigned using predefined percentages of the maximum pixel intensity from the CMR images, and these values were color-coded onto the typical color scheme used in the EAM system. Ablation was then targeted at heterogeneous tissue channels at the interface of the scar core and scar border zone that had correlated EAM abnormalities. Patients in the image-guided group had lower RF lesion times overall, with a higher non-inducible proportion after substrate ablation. In addition, image-guided ablation patients had more improved VT-free survival compared to those undergoing traditional ablation strategies. Interestingly, in this study, patients with heterogeneous tissue channels that did not correlate with EAM abnormalities and thus were not targeted had increased risks of recurrent ventricular arrhythmia events, suggesting that delayed-enhancement cardiac MRI when integrated with EAM may be more sensitive for characterizing the arrhythmogenic substrate.

In a similar prospective non-randomized study by Zghaib et al., patients were assigned to MRI-derived scar-guided ablation or traditional ablation.^66^ Those patients undergoing imaging-guided ablation had their LGE-MRI sequences processed to identify areas of increased signal intensity using a >6 standard deviation approach above the normal myocardium approach. Scar contours were imported and registered with the EAM system using 3 fixed reference points (LV apex, aorta, and mitral annulus). Areas of LGE were evaluated for abnormal electrograms (EGMs). Regions of abnormal EGMs and abnormal LGE were then targeted for ablation. While acute success, defined as non-inducibility or clinical VT termination, was achieved in all patients, patients in the imaging-guided ablation group had a lower incidence of recurrence than those in the standard ablation group. This was in the setting of a lower LV ejection fraction and a higher number of prior ablations, though these differences in baseline characteristics did not reach statistical significance.

**Figure 7:**
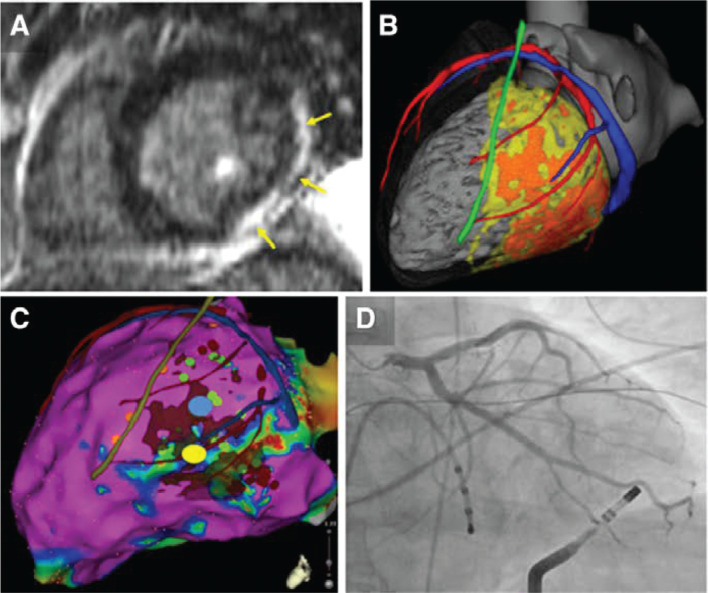
Example of image integration to assist ventricular tachycardia ablation in a case of myocarditis. **A:** Lateral and inferior left ventricular scar on cardiac magnetic resonance (CMR) (arrows). **B:** Patient-specific 3-dimensional model built from merged computed tomography (CT) (anatomy) and magnetic resonance imaging (scar) data. Cardiac chambers (gray), coronary arteries, and veins (in red and blue, respectively), left phrenic nerve (green), as segmented from CT, and dense scar and gray zone (in orange and yellow, respectively) as segmented from CMR. **C:** Epicardial bipolar voltage map with merged imaging model. Mid-diastolic potentials (not shown) are recorded during VT on an epicardial lateral left ventricular site (yellow dot in **C**). This potential target for epicardial ablation is far enough from the left phrenic nerve path derived from imaging (green line in **C**), which accurately matches sites of phrenic capture (orange dots in **C**). However, CT demonstrates the proximity of this site to a marginal branch of the circumflex artery on the registered imaging model. Confirmatory coronary angiography (**D**) demonstrates contact between the tip of the ablation catheter and the coronary artery. Reprinted with permission from Mahida S, Sacher F, Dubois R, et al. Cardiac imaging in patients with ventricular tachycardia. *Circulation*. 2017;136(25):2491–2507.

Cardiac CT is particularly useful in helping in the structural assessment of patients undergoing VT ablation. Cardiac CT can be used to delineate epicardial fat, coronary anatomy, and the course of the phrenic vein, as seen in **Figure 7**.^67^ Registration of the cardiac CT with the electroanatomic map can allow the proceduralist to determine whether or not RF ablation is feasible and safe, in particular in the epicardium, where overlying epicardial fat may insulate the substrate, or epicardial coronary arteries or the phrenic nerve make RF energy delivery unsafe.

Intraprocedural real-time MRI is an experimental technique in development largely in non-human models.^68^ The advancement of this technology is limited by the need for non-ferromagnetic mapping and ablation equipment. In addition, the lack of MRI-safe defibrillator systems limits the safety of performing VT ablation with patients inside an MRI scanner. The limited human studies that have been performed have been mostly feasibility and safety studies in patients undergoing simple ablation for typical atrial flutter.^69^ However, the relative advantage of real-time MRI would most likely be best leveraged in the complex ablation population and, in particular, in the VT ablation population, as understanding real-time substrate characteristics and lesion extent in relatively thick myocardium is key in abolishing potential VT circuits. The authors of this review anticipate growth over the next decade in this field, but clinical applications at this time are still in their infancy and limited to research purposes.

ICE is commonly used during the ablation of ventricular arrhythmias. It is particularly useful in understanding catheter-contact issues when interacting with the complex geometry of the papillary muscles in the LV and ventricular trabeculations in the right ventricle (RV) **(Figure 4B)**. In addition, differences in the echogenicity of the myocardium on ICE evaluation can suggest possible areas of scarring and thus targets for ablation in the mid-myocardium or epicardium.^70^ ICE also serves as an important safety tool in assessing for pericardial effusion and gauging ablation lesion depth. In a registry analysis, the use of ICE has been associated with decreased VT-related readmission and the need for repeat VT ablation.^71^ Small observational cohorts also suggest that ICE may decrease the likelihood of pericardial tamponade.^72^

Beyond adjunctive imaging modalities, significant interest has begun to develop around the radiological treatment of ventricular arrhythmias in patients with VT refractory to catheter ablation. Non-invasive cardiac ablation with stereotactic body radiation therapy (SBRT) as a treatment modality has been performed in a series of small investigative studies and appears to have significant promise.^73,74^ Methods for the identification of radioablation target volumes vary but can involve integrating multiple non-invasive and invasive techniques. In a previous study, electrocardiographic imaging using a large number of body surface electrodes was performed during VT induced by non-invasive programmed stimulation through an implantable cardioverter-defibrillator. Isochronal maps, repolarization patterns, and models of epicardial potentials are constructed from body surface potentials integrated with anatomical data from cardiac CT. These maps, in conjunction with traditional preprocedural imaging for VT, as described in the earlier sections, are used to build contoured volume targets for radioablation. A single-fraction dose of 25 Gy is then typically delivered to the target volume. Studies of SBRT, thus far, have demonstrated a good safety profile in the mid-term as well as a significant reduction in VT episodes.^73,74^ However, there is significant non-arrhythmic mortality in the small cohort of patients selected, and long-term efficacy and appropriate patient selection criteria for this treatment modality still require further investigation.

### Postprocedural imaging

Postprocedural imaging for the evaluation of VT has not yet reached wide clinical utilization as a routine evaluation. In a study evaluating postinfarction VT ablation patients referred for repeat VT ablation, a “dark core” was seen on LGE images, which correlated well with areas of ablation lesion delivery.^75^ This was seen in MRI scans remote from the initial ablation lesions (on average 30 months after ablation) and in contrast to prior animal model studies and small human studies of ablation lesions in idiopathic ventricular arrhythmias, which have shown microvascular obstruction patterns and surrounding edema in the early phase followed by typical increased LGE intensity in the chronic phase.^76^ This is a potentially promising breakthrough in postprocedural lesion analysis as this could show potential inadequate substrate homogenization and guide future ablation targets in patients with recurrent VT.

## Conclusion and future directions

An ever-growing array of imaging modalities are available to aid in improving the safety and efficacy of catheter ablation. Cardiac CT, cardiac MRI, and ICE are particularly useful and have been shown to improve outcomes in catheter ablation. Future development of these technologies will allow better real-time integration with EAM systems and high-resolution imaging of the myocardial substrate. These advances will allow for shorter procedural times, safer risk profiles for both the patients and the operators, and improved efficacy in targeting the true substrate of interest.

## References

[1] Joseph JP, Rajappan K (2012). Radiofrequency ablation of cardiac arrhythmias: past, present and future. QJM Int J Med.

[2] Scheinman MA, Rutherford JD (2017). The development of cardiac arrhythmia ablation. Circulation.

[3] Lam WC, Pennell DJ (2016). Imaging of the heart: historical perspective and recent advances. Postgrad Med J.

[4] Hindricks G, Potpara T, Dagres N (2021). 2020 ESC Guidelines for the diagnosis and management of atrial fibrillation developed in collaboration with the European Association for Cardio-Thoracic Surgery (EACTS): the Task Force for the diagnosis and management of atrial fibrillation of the European Society of Cardiology (ESC) Developed with the special contribution of the European Heart Rhythm Association (EHRA) of the ESC. Eur Heart J.

[5] January CT, Wann LS, Alpert JS (2014). 2014 AHA/ACC/HRS guideline for the management of patients with atrial fibrillation: executive summary: a report of the American College of Cardiology/American Heart Association Task Force on practice guidelines and the Heart Rhythm Society. Circulation.

[6] Manning WJ, Weintraub RM, Waksmonski CA (1995). Accuracy of transesophageal echocardiography for identifying left atrial thrombi. A prospective, intraoperative study. Ann Intern Med.

[7] Hwang JJ, Chen JJ, Lin SC (1993). Diagnostic accuracy of transesophageal echocardiography for detecting left atrial thrombi in patients with rheumatic heart disease having undergone mitral valve operations. Am J Cardiol.

[8] Romero J, Husain SA, Kelesidis I, Sanz J, Medina HM, Garcia MJ (2013). Detection of left atrial appendage thrombus by cardiac computed tomography in patients with atrial fibrillation: a meta-analysis. Circ Cardiovasc Imaging.

[9] Chen J, Zhang H, Zhu D, Wang Y, Byanju S, Liao M (2019). Cardiac MRI for detecting left atrial/left atrial appendage thrombus in patients with atrial fibrillation : meta-analysis and systematic review. Herz.

[10] Kitkungvan D, Nabi F, Ghosn MG (2016). Detection of LA and LAA thrombus by CMR in patients referred for pulmonary vein isolation. JACC Cardiovasc Imaging.

[11] Beiert T, Lodde PC, Linneborn LPT (2018). Outcome in patients with left common pulmonary vein after cryoablation with second-generation cryoballoon. Pacing Clin Electrophysiol.

[12] den Uijl DW, Tops LF, Delgado V (2011). Effect of pulmonary vein anatomy and left atrial dimensions on outcome of circumferential radiofrequency catheter ablation for atrial fibrillation. Am J Cardiol.

[13] Khoueiry Z, Albenque J-P, Providencia R (2016). Outcomes after cryoablation vs. radiofrequency in patients with paroxysmal atrial fibrillation: impact of pulmonary veins anatomy. Europace.

[14] Linhart M, Lewalter T, Mittmann-Braun EL (2013). Left atrial pressure as predictor for recurrence of atrial fibrillation after pulmonary vein isolation. J Interv Card Electrophysiol.

[15] Kawel-Boehm N, Hetzel SJ, Ambale-Venkatesh B (2020). Reference ranges (“normal values”) for cardiovascular magnetic resonance (CMR) in adults and children: 2020 update. J Cardiovasc Magn Reson.

[16] Stojanovska J, Cronin P, Patel S (2011). Reference normal absolute and indexed values from ECG-gated MDCT: left atrial volume, function, and diameter. Am J Roentgenol.

[17] Abecasis J, Dourado R, Ferreira A (2009). Left atrial volume calculated by multi-detector computed tomography may predict successful pulmonary vein isolation in catheter ablation of atrial fibrillation. Europace.

[18] Montefusco A, Biasco L, Blandino A (2010). Left atrial volume at MRI is the main determinant of outcome after pulmonary vein isolation plus linear lesion ablation for paroxysmal-persistent atrial fibrillation. J Cardiovasc Med (Hagerstown).

[19] Tops LF, van der Wall EE, Schalij MJ, Bax JJ (2007). Multi-modality imaging to assess left atrial size, anatomy and function. Heart.

[20] Ikenouchi T, Inaba O, Takamiya T (2021). The impact of left atrium size on selection of the pulmonary vein isolation method for atrial fibrillation: cryoballoon or radiofrequency catheter ablation. Am Heart J.

[21] Marrouche NF, Wilber D, Hindricks G (2014). Association of atrial tissue fibrosis identified by delayed enhancement MRI and atrial fibrillation catheter ablation: the DECAAF study. JAMA.

[22] Khurram IM, Habibi M, Gucuk Ipek E (2016). Left atrial LGE and arrhythmia recurrence following pulmonary vein isolation for paroxysmal and persistent AF. JACC Cardiovasc Imaging.

[23] Chelu MG, King JB, Kholmovski EG (2018). Atrial fibrosis by late gadolinium enhancement magnetic resonance imaging and catheter ablation of atrial fibrillation: 5-year follow-up data. J Am Heart Assoc.

[24] Kholmovski EG, Morris AK, Chelu MG (2019). Cardiac MRI and fibrosis quantification. Card Electrophysiol Clin.

[25] Shade JK, Ali RL, Basile D (2020). Preprocedure application of machine learning and mechanistic simulations predicts likelihood of paroxysmal atrial fibrillation recurrence following pulmonary vein isolation. Circ Arrhythm Electrophysiol.

[26] Boyle PM, Zghaib T, Zahid S (2019). Computationally guided personalized targeted ablation of persistent atrial fibrillation. Nat Biomed Eng.

[27] Bertaglia E, Bella PD, Tondo C (2009). Image integration increases efficacy of paroxysmal atrial fibrillation catheter ablation: results from the CartoMerge Italian Registry. Europace.

[28] Caponi D, Corleto A, Scaglione M (2010). Ablation of atrial fibrillation: does the addition of three-dimensional magnetic resonance imaging of the left atrium to electroanatomic mapping improve the clinical outcome? A randomized comparison of Carto-Merge vs. Carto-XP three-dimensional mapping ablation in patients with paroxysmal and persistent atrial fibrillation. Europace.

[29] Gianni C, Della Rocca DG, Horton RP, Burkhardt JD, Natale A, Al-Ahmad A (2021). Real-time 3D intracardiac echocardiography. Card Electrophysiol Clin.

[30] Romero J, Patel K, Briceno D (2020). Fluoroless atrial fibrillation catheter ablation: technique and clinical outcomes. Card Electrophysiol Clin.

[31] Lerman BB, Markowitz SM, Liu CF, Thomas G, Ip JE, Cheung JW (2017). Fluoroless catheter ablation of atrial fibrillation. Heart Rhythm.

[32] Bulava A, Hanis J, Eisenberger M (2015). Catheter ablation of atrial fibrillation using zero-fluoroscopy technique: a randomized trial. Pacing Clin Electrophysiol.

[33] Baykaner T, Quadros KK, Thosani A (2020). Safety and efficacy of zero fluoroscopy transseptal puncture with different approaches. Pacing Clin Electrophysiol.

[34] Zei PC, Quadros KK, Clopton P (2020). Safety and efficacy of minimal- versus zero-fluoroscopy radiofrequency catheter ablation for atrial fibrillation: a multicenter, prospective study. J Innov Card Rhythm Manag.

[35] Sommer P, Bertagnolli L, Kircher S (2018). Safety profile of near-zero fluoroscopy atrial fibrillation ablation with non-fluoroscopic catheter visualization: experience from 1000 consecutive procedures. Europace.

[36] Matsubara TJ, Fujiu K, Shimizu Y (2020). Fluoroless and contrast-free catheter ablation without a lead apron in routine clinical practice. Sci Rep.

[37] Alyesh D, Venkataraman G, Stucky A, Joyner J, Choe W, Sundaram S (2021). Acute safety and efficacy of fluoroless cryoballoon ablation for atrial fibrillation. J Innov Card Rhythm Manag.

[38] Fochler F, Yamaguchi T, Kheirkahan M, Kholmovski EG, Morris AK, Marrouche NF (2019). Late gadolinium enhancement magnetic resonance imaging guided treatment of post-atrial fibrillation ablation recurrent arrhythmia. Circ Arrhythm Electrophysiol.

[39] Rostamian A, Narayan SM, Thomson L, Fishbein M, Siegel RJ (2014). The incidence, diagnosis, and management of pulmonary vein stenosis as a complication of atrial fibrillation ablation. J Interv Card Electrophysiol.

[40] Shimizu Y, Yoshitani K, Murotani K (2018). The deeper the pouch is, the longer the radiofrequency duration and higher the radiofrequency energy needed-Cavotricuspid isthmus ablation using intracardiac echocardiography. J Arrhythmia.

[41] Scaglione M, Caponi D, Di Donna P (2004). Typical atrial flutter ablation outcome: correlation with isthmus anatomy using intracardiac echo 3D reconstruction. Europace.

[42] Herman D, Osmancik P, Zdarska J, Prochazkova R (2017). Routine use of intracardiac echocardiography for atrial flutter ablation is associated with reduced fluoroscopy time, but not with a reduction of radiofrequency energy delivery time. J Atr Fibrillation.

[43] Yalagudri S, Zin Thu N, Devidutta S (2017). Tailored approach for management of ventricular tachycardia in cardiac sarcoidosis. J Cardiovasc Electrophysiol.

[44] Muser D, Santangeli P, Selvanayagam JB, Nucifora G (2019). Role of cardiac magnetic resonance imaging in patients with idiopathic ventricular arrhythmias. Curr Cardiol Rev.

[45] Reithmann C, Kling T, Herkommer B, Fiek M, Ulbrich M (2019). Magnetic resonance imaging abnormalities in the basal interventricular septum of patients with left ventricular outflow tract arrhythmias. J Cardiovasc Electrophysiol.

[46] Dickfeld T, Tian J, Ahmad G (2011). MRI-guided ventricular tachycardia ablation: integration of late gadolinium-enhanced 3D scar in patients with implantable cardioverter-defibrillators. Circ Arrhythm Electrophysiol.

[47] Siontis KC, Kim HM, Sharaf Dabbagh G (2017). Association of preprocedural cardiac magnetic resonance imaging with outcomes of ventricular tachycardia ablation in patients with idiopathic dilated cardiomyopathy. Heart Rhythm.

[48] Hendriks AA, Kis Z, Glisic M, Bramer WM, Szili-Torok T (2020). Pre-procedural image-guided versus non-image-guided ventricular tachycardia ablation – a review. Neth Heart J.

[49] Ávila P, Pérez-David E, Izquierdo M (2015). Scar extension measured by magnetic resonance-based signal intensity mapping predicts ventricular tachycardia recurrence after substrate ablation in patients with previous myocardial infarction. JACC Clin Electrophysiol.

[50] Piers SRD, Tao Q, van Huls van Taxis CFB, Schalij MJ, van der Geest RJ, Zeppenfeld K (2013). Contrast-enhanced MRI-derived scar patterns and associated ventricular tachycardias in nonischemic cardiomyopathy: implications for the ablation strategy. Circ Arrhythm Electrophysiol.

[51] Oloriz T, Silberbauer J, Maccabelli G (2014). Catheter ablation of ventricular arrhythmia in nonischemic cardiomyopathy: anteroseptal versus inferolateral scar sub-types. Circ Arrhythm Electrophysiol.

[52] Kuo L, Liang JJ, Nazarian S, Marchlinski FE (2020). Multimodality imaging to guide ventricular tachycardia ablation in patients with non-ischaemic cardiomyopathy. Arrhythmia Electrophysiol Rev.

[53] Andreu D, Ortiz-Pérez JT, Boussy T (2014). Usefulness of contrast-enhanced cardiac magnetic resonance in identifying the ventricular arrhythmia substrate and the approach needed for ablation. Eur Heart J.

[54] Njeim M, Yokokawa M, Frank L (2016). Value of cardiac magnetic resonance imaging in patients with failed ablation procedures for ventricular tachycardia. J Cardiovasc Electrophysiol.

[55] Kuo L, Liang JJ, Han Y (2020). Association of septal late gadolinium enhancement on cardiac magnetic resonance with ventricular tachycardia ablation targets in nonischemic cardiomyopathy. J Cardiovasc Electrophysiol.

[56] Nishimura T, Patel HN, Wang S (2021). Prognostic value of cardiac magnetic resonance septal late gadolinium enhancement patterns for periaortic ventricular tachycardia ablation: heterogeneity of the anteroseptal substrate in nonischemic cardiomyopathy. Heart Rhythm.

[57] Perez-David E, Arenal A, Rubio-Guivernau JL (2011). Noninvasive identification of ventricular tachycardia-related conducting channels using contrast-enhanced magnetic resonance imaging in patients with chronic myocardial infarction: comparison of signal intensity scar mapping and endocardial voltage mapping. J Am Coll Cardiol.

[58] Fernández-Armenta J, Berruezo A, Andreu D (2013). Three-dimensional architecture of scar and conducting channels based on high resolution ce-CMR: insights for ventricular tachycardia ablation. Circ Arrhythm Electrophysiol.

[59] Piers SRD, Tao Q, de Riva Silva M (2014). CMR-based identification of critical isthmus sites of ischemic and nonischemic ventricular tachycardia. JACC Cardiovasc Imaging.

[60] Peretto G, Sala S, Basso C (2020). Inflammation as a predictor of recurrent ventricular tachycardia after ablation in patients with myocarditis. J Am Coll Cardiol.

[61] Imanli H, Ume KL, Jeudy J (2019). Ventricular tachycardia (VT) substrate characteristics: insights from multimodality structural and functional imaging of the vt substrate using cardiac MRI scar, 123I-metaiodobenzylguanidine SPECT innervation, and bipolar voltage. J Nucl Med.

[62] Roca-Luque I, Rivas-Gándara N, Francisco-Pascual J (2019). Preprocedural imaging to guide transcoronary ethanol ablation for refractory septal ventricular tachycardia. J Cardiovasc Electrophysiol.

[63] Trayanova NA, Pashakhanloo F, Wu KC, Halperin HR (2017). Imaging-based simulations for predicting sudden death and guiding ventricular tachycardia ablation. Circ Arrhythm Electrophysiol.

[64] Ashikaga H, Arevalo H, Vadakkumpadan F (2013). Feasibility of image-based simulation to estimate ablation target in human ventricular arrhythmia. Heart Rhythm.

[65] Andreu D, Penela D, Acosta J (2017). Cardiac magnetic resonance-aided scar dechanneling: influence on acute and long-term outcomes. Heart Rhythm.

[66] Zghaib T, Ipek EG, Hansford R (2018). Standard ablation versus magnetic resonance imaging-guided ablation in the treatment of ventricular tachycardia. Circ Arrhythm Electrophysiol.

[67] Conte E, Mushtaq S, Carbucicchio C (2021). State of the art paper: cardiovascular CT for planning ventricular tachycardia ablation procedures. J Cardiovasc Comput Tomogr.

[68] Mukherjee RK, Chubb H, Roujol S, Razavi R, O’Neill MD (2019). Advances in real-time MRI-guided electrophysiology. Curr Cardiovasc Imaging Rep.

[69] Paetsch I, Sommer P, Jahnke C (2019). Clinical workflow and applicability of electrophysiological cardiovascular magnetic resonance-guided radiofrequency ablation of isthmus-dependent atrial flutter. Eur Heart J Cardiovasc Imaging.

[70] Bala R, Ren J-F, Hutchinson MD (2011). Assessing epicardial substrate using intracardiac echocardiography during VT ablation. Circ Arrhythm Electrophysiol.

[71] Field ME, Gold MR, Reynolds MR (2020). Real-world outcomes of ventricular tachycardia catheter ablation with versus without intracardiac echocardiography. J Cardiovasc Electrophysiol.

[72] Kitamura T, Nakajima M, Kawamura I (2021). Safety and effectiveness of intracardiac echocardiography in ventricular tachycardia ablation: a nationwide observational study. Heart Vessels.

[73] Cuculich PS, Schill MR, Kashani R (2017). Noninvasive cardiac radiation for ablation of ventricular tachycardia. N Engl J Med.

[74] Neuwirth R, Cvek J, Knybel L (2019). Stereotactic radiosurgery for ablation of ventricular tachycardia. Europace.

[75] Dabbagh GS, Ghannam M, Siontis KC (2021). Magnetic resonance mapping of catheter ablation lesions after post-infarction ventricular tachycardia ablation. JACC Cardiovasc Imaging.

[76] Dickfeld T, Vunnam R (2021). Chronic ablation lesions on CMR: is black a red herring?. JACC Cardiovasc Imaging.

